# Long noncoding RNA *TTN-AS1* enhances the malignant characteristics of osteosarcoma by acting as a competing endogenous RNA on microRNA-376a thereby upregulating dickkopf-1

**DOI:** 10.18632/aging.102280

**Published:** 2019-09-16

**Authors:** Shenglong Li, Fei Liu, Yi Pei, Wei Wang, Ke Zheng, Xiaojing Zhang

**Affiliations:** 1 Department of Bone and Soft Tissue Tumor Surgery, Cancer Hospital of China Medical University, Liaoning Cancer Hospital and Institute, Shenyang 110042, Liaoning, P.R. China

**Keywords:** TTN antisense RNA 1, dickkopf-1, microRNA-376a, therapeutic target

## Abstract

The expression levels and detailed functions of *TTN-AS1* in osteosarcoma (OS) have not yet been explored. This study aimed to measure *TTN-AS1* expression in OS tissues and cell lines, investigate its specific roles in the aggressive characteristics of OS cells in vitro and in vivo, and elucidate the regulatory mechanisms of *TTN-AS1* action. *TTN-AS1* expression was high in OS tissue samples and cell lines; *TTN-AS1* overexpression correlated with the clinical stage, distant metastasis, and shorter overall survival of the patients. A *TTN-AS1* knockdown inhibited OS cell proliferation, migration, and invasion and induced apoptosis in vitro and slowed tumor growth in vivo. Mechanism investigation revealed that *TTN-AS1* acts as a competing endogenous RNA on microRNA-376a-3p (miR-376a) in OS cells. Dickkopf-1 (*DKK1*) mRNA was identified as a direct target of miR-376a in OS cells. Resumption of *DKK1* expression reversed the tumor-suppressive activities of miR-376a overexpression in OS cells. The knockdown of miR-376a counteracted the reduction in the malignant characteristics of OS cells by the downregulation of *TTN-AS1*. In conclusion, *TTN-AS1* functions as a competing endogenous RNA targeting miR-376a and increases the malignancy of OS cells in vitro and in vivo by upregulating DKK1.

## INTRODUCTION

Osteosarcoma (OS) is a highly malignant bone cancer and mainly affects children and adolescents [1]. It frequently occurs in a long bone of a limb and the growth plate of a metaphysis [2]. Several risk factors, including alkylating agents, ionizing radiation, and hereditary retinoblastoma, have been reported to promote the initiation of OS [3]. At present, adjuvant therapy, surgical resection, and postoperative chemotherapy are the primary therapeutic modalities for patients with OS; however, the rates of death and metastasis remain high despite the effectiveness of the above approaches [4]. Patients with OS without any local or distant metastasis have a 5-year survival rate of 60%–70%, whereas the 5-year survival rate of patients with OS metastasis is markedly lower, i.e., only <30% [5]. Therefore, comprehensive identification of the mechanisms underlying OS initiation and progression is required for the development of promising therapeutic strategies and improvement of clinical outcomes.

Long noncoding RNAs (lncRNAs) are defined as protein-non-coding transcripts greater than 200 nucleotides in length [6]. Increasing evidence has revealed the important regulatory effects of lncRNAs on various parameters of physiological and pathological processes, especially cancer [7, 8]. In OS, thousands of lncRNAs are differentially expressed and implicated in pathogenesis through various mechanisms: by serving as RNA decoys as well as participating in alternative splicing and epigenetic, transcriptional, and post-transcriptional modifications [9–11]. LncRNAs overexpressed in OS act as oncogenic RNAs, whereas underexpressed lncRNAs have tumor-suppressive effects [12–14]. Accordingly, research on OS-related lncRNAs and the elucidation of their mechanisms of action may reveal potential targets for the diagnosis, prognosis, prevention, and/or treatment of OS. Nevertheless, in comparison with the lncRNAs with known functions in OS, there is a large number of lncRNAs that possibly contribute to the formation and progression of OS but have not yet been clearly identified; these lncRNAs are yet to be studied in-depth.

MicroRNAs (miRNAs) are a group of noncoding short regulatory RNAs, 18–25 nucleotides long [15]. MiRNAs can recognize the 3′-untranslated region (3′-UTR) of target mRNAs and inhibit their expression by suppressing translation and/or promoting mRNA degradation [16]. MiRNAs exert crucial actions in a variety of cellular and molecular biological processes, including carcinogenesis and cancer progression [17]. Changes in the expression of miRNAs are prevalent in almost all human cancer types [18–20]. As for OS, many miRNAs are dysregulated, and their dysregulation serves as a crucial contributing factor for the aggressive characteristics of OS [21–23]. Therefore, a miRNA-based targeted therapy may be a promising strategy against OS.

In recent years, an lncRNA called TTN antisense RNA 1 (*TTN-AS1*) was reported to be overexpressed and play oncogenic roles in cervical cancer [24], papillary thyroid cancer [25], gastric cancer [26], hepatocellular carcinoma [27], esophageal squamous cell carcinoma [28], and lung adenocarcinoma [29, 30]. Nevertheless, the expression levels and detailed participation of *TTN-AS1* in OS have not yet been studied. Herein, we attempted to assess *TTN-AS1* expression in OS tumor samples and cell lines to investigate its specific roles in the aggressiveness of OS cells in vitro and in vivo and elucidate its regulatory mechanisms of action.

## RESULTS

### Upregulation of *TTN-AS1* is associated with poor clinical outcomes among patients with OS

**Figure 1:**
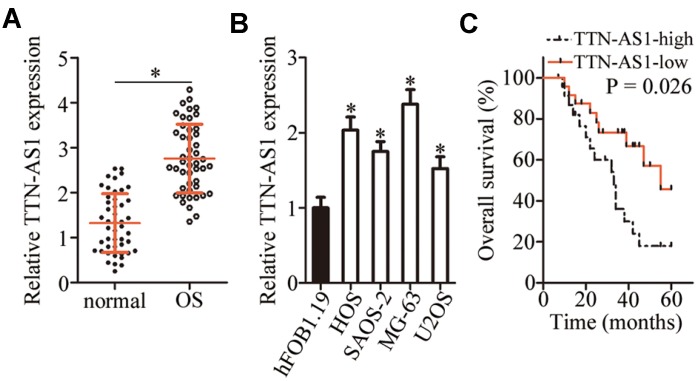
***TTN-AS1* is overexpressed in OS tissue samples and cell lines.** (**A**) The expression of *TTN-AS1* was analyzed in 47 pairs of OS tissue samples and adjacent normal bone tissues using RT-qPCR. *P < 0.05 vs. the normal bone tissues. (**B**) RT-qPCR was performed to determine *TTN-AS1* expression in four OS cell lines (HOS, SAOS-2, MG-63, and U2OS) and normal osteoblasts (hFOB1.19 cells). *P < 0.05 vs. hFOB1.19 cells. (**C**) The Kaplan–Meier survival analysis and logrank test were applied to assess the relation between *TTN-AS1* levels and the overall survival of patients with OS. The median value of *TTN-AS1* expression among the OS tissue samples was chosen as a cutoff. P = 0.026.

To determine the specific role of *TTN-AS1* in OS, the expression profile of this lncRNA was examined in 47 pairs of OS tissue samples and adjacent-normal-bone tissue samples. *TTN-AS1* was found to be overexpressed in the OS tissue samples relative to the adjacent normal bone tissues, as revealed by reverse-transcription quantitative PCR (RT-qPCR; Figure 1A, P < 0.05). Additionally, the expression of *TTN-AS1* was quantified in a panel of OS cell lines (HOS, SAOS-2, MG-63, and U2OS) and in normal osteoblasts (hFOB1.19 cells). The results showed that *TTN-AS1* expression was higher in the four tested OS cell lines than in hFOB1.19 cells (Figure 1B, P < 0.05).

To assess the clinical value of *TTN-AS1*, we first analyzed its correlation with clinical parameters among the patients with OS. The median value of *TTN-AS1* expression in OS tissue samples was chosen as the cutoff and, on this basis, all the patients with OS were assigned to either the *TTN- AS1* low-expression group or *TTN-AS1* high-expression group. The high level of *TTN-AS1* manifested a significant association with the clinical stage (P = 0.015; Table 1) and distant metastasis (P = 0.017; Table 1). Notably, patients with OS overexpressing *TTN-AS1* showed shorter overall survival than the patients with OS underexpressing *TTN-AS1* (Figure 1C, P = 0.026). These results implied that *TTN-AS1* may be closely associated with the pathogenesis of OS.

**Table 1:** The correlation between TTN-AS1 expression level and clinicopathological parameters of patients with osteosarcoma.

**Parameters**	**TTN-AS1 expression**		**P value**
	**High (n=24)**	**Low (n=23)**	
**Age (years)**			0.724
< 18	18	19	
≥18	6	4	
**Gender**			0.556
Male	13	15	
Female	11	8	
**Tumor size (cm)**			0.380
< 5	16	12	
≥ 5	8	11	
**Clinical staging**			0.015*
I-II	11	19	
III	13	4	
**Distant metastasis**			0.017*
Absence	14	21	
Presence	10	2	

### A reduction in *TTN-AS1* expression inhibits the malignant characteristics of OS cells in vitro

**Figure 2:**
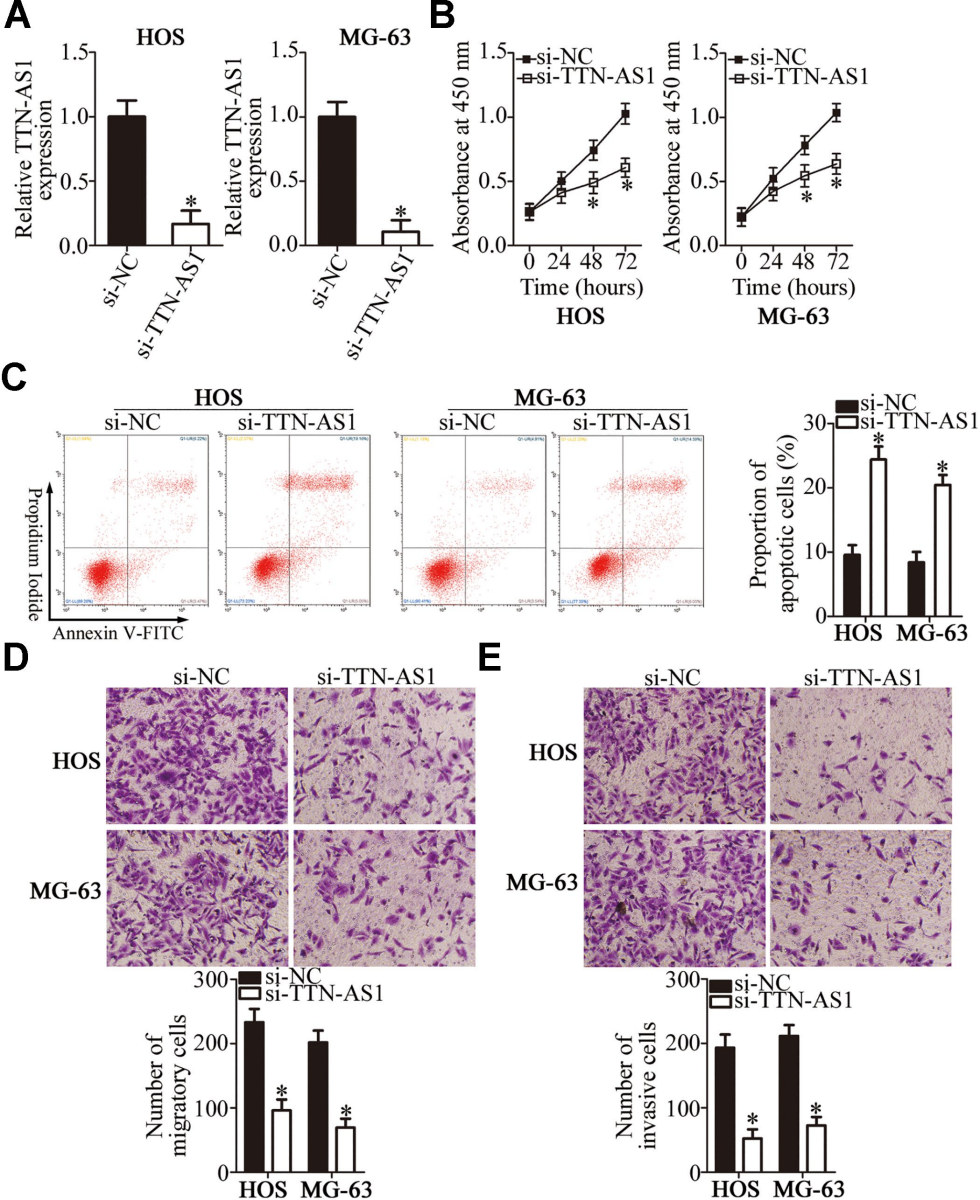
**The *TTN-AS1* knockdown suppresses the proliferation, migration, and invasiveness but promotes the apoptosis of HOS and MG-63 cells.** (**A**) HOS and MG-63 cells were transfected with either si-TTN-AS1 or si-NC. At 48 h post-transfection, the cells were collected and, then, subjected to RT-qPCR analysis for transfection efficiency evaluation. *P < 0.05 vs. the si-NC group. (**B**) The CCK-8 assay was conducted to assess cellular proliferation after 0, 24, 48, and 72 h of cultivation of si-TTN-AS1-transfected or si-NC-transfected HOS and MG-63 cells. *P < 0.05 vs. group si-NC. (**C**) Flow cytometry was performed to determine the apoptotic rate of HOS and MG-63 cells after transfection with either si-TTN-AS1 or si-NC. *P < 0.05 vs. the si-NC group. (**D**, **E**) The migratory and invasive abilities of *TTN-AS1*–deficient HOS and MG-63 cells were analyzed in Transwell migration and invasion assays. *P < 0.05 vs. group si-NC.

Having detected the aberrant upregulation of *TTN-AS1* in OS, we next attempted to determine the functions of *TTN-AS1* in OS progression. Cell lines HOS and MG-63 showed higher *TTN-AS1* expression than the other two OS cell lines; accordingly, HOS and MG-63 cells were chosen for subsequent experiments and were transfected with either a small interfering RNA [siRNA] against *TTN-AS1* (si-TTN-AS1) or a negative control siRNA (si-NC). *TTN-AS1* was successfully knocked down in HOS and MG-63 cells after transfection of si-TTN-AS1 (Figure 2A, P < 0.05). A Cell Counting Kit-8 (CCK-8) assay was performed to evaluate the influence of *TTN-AS1* on OS cell proliferation. The si-TTN-AS1 transfection obviously reduced the proliferative ability of HOS and MG-63 cells compared with that in the si-NC group (Figure 2B, P < 0.05). Then, flow cytometric analysis was conducted to test whether si-TTN-AS1 introduction increases OS cell apoptosis. As expected, the proportion of apoptotic cells was greater among HOS and MG-63 cells after transfection with si-TTN-AS1 (Figure 2C, P < 0.05). In addition, Transwell migration and invasion assays revealed that the *TTN-AS1* knockdown notably reduced the migration (Figure 2D, P < 0.05) and invasiveness (Figure 2E, P < 0.05) of HOS and MG-63 cells. In general, these findings suggested that the *TTN-AS1* downregulation slowed the malignant progression of OS in vitro.

### *TTN-AS1* acts as a competing endogenous RNA (ceRNA) on miR-376a-3p (miR-376a) in OS cells

**Figure 3:**
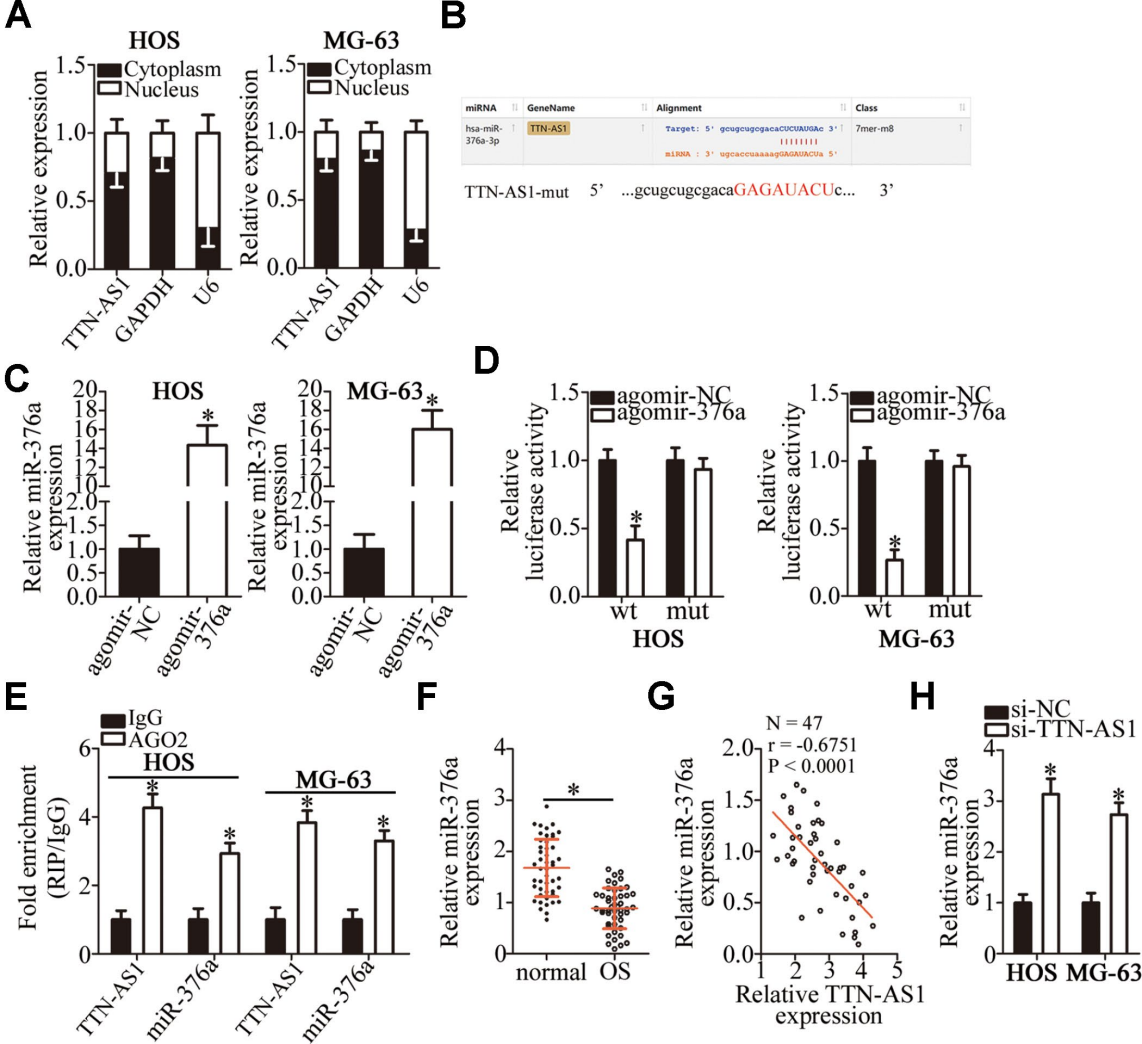
***TTN-AS1* functions as a ceRNA for miR-376a in OS cells.** (**A**) The distribution of *TTN-AS1* within OS cells was determined by the nuclear/cytoplasmic fractionation assay. (**B**) The wild-type miR-376a-binding sequences in *TTN-AS1*, as predicted by starBase 3.0. The mutations in the *TTN-AS1* sequence that disrupt the interaction between *TTN-AS1* and miR-376a are shown too. (**C**) HOS and MG-63 cells that were transfected with either agomir-376a or agomir-NC were harvested and analyzed for miR-376a expression by RT-qPCR. *P < 0.05 vs. the agomir-NC group. (**D**) Luciferase reporter assays were performed on HOS and MG-63 cells that were transfected with either agomir-376a or agomir-NC and either TTN-AS1-wt or TTN-AS1-mut. *P < 0.05 vs. group agomir-NC. (**E**) The RIP assay was conducted to assess the direct interaction between *TTN-AS1* and miR-376a. *P < 0.05 vs. the IgG group. (**F**) The expression profile of miR-376a in the 47 pairs of OS tissues and adjacent-normal-bone tissue samples was analyzed by RT-qPCR. *P < 0.05 vs. the normal bone tissues. (**G**) An inverse correlation between *TTN-AS1* and miR-376a expression levels was validated in the OS tissue samples by Spearman’s correlation analysis. r = -0.6751, P < 0.0001. (**H**) Expression of miR-376a in HOS and MG-63 cells transfected with either si-TTN-AS1 or si-NC was determined by RT-qPCR. *P < 0.05 vs. the si-NC group.

To investigate the molecular events involved in *TTN-AS1*–mediated OS progression, a nuclear/cytoplasmic fractionation assay was conducted to determine the distribution of *TTN-AS1* inside OS cells. The data indicated that *TTN-AS1* is mainly located in the cytoplasm of OS cells (Figure 3A); this finding suggested that this lncRNA may serve as a ceRNA for some miRNA(s) [31]. Herein, the candidate miRNAs that could be inactivated by *TTN-AS1* were predicted via starBase 3.0. The results indicated that *TTN-AS1* contains one conserved binding site for miR-376a (Figure 3B). Luciferase reporter and RNA immunoprecipitation (RIP) assays were performed to further characterize the relation between *TTN-AS1* and miR-376a in OS cells. MiRNA-376a agomir (agomir-376a) transfection-mediated upregulation of miR-376a (Figure 3C, P < 0.05) noticeably decreased the luciferase activity of the TTN-AS1-wt plasmid (the plasmid expressing *TTN-AS1* containing the wild-type binding site for miR-376a; P < 0.05); however, the luciferase activity of TTN-AS1-mut (the plasmid expressing *TTN-AS1* containing a mutant binding site for miR-376a) was unaffected in both HOS and MG-63 cells when miR-376a was overexpressed, as evidenced by the luciferase reporter assay (Figure 3D). In addition, the findings of the RIP assay indicated that *TTN-AS1* and miR-376a were preferentially enriched by an anti-AGO2 antibody after immunoprecipitation in the lysates of HOS and MG-63 cells (Figure 3E, P < 0.05). This finding suggested that miR-376a is a target of *TTN-AS1* in OS cells. MiR-376a expression was subsequently measured in the 47 pairs of OS tissue samples and adjacent normal bone tissues by RT-qPCR. MiR-376a was found to be significantly underexpressed in the OS tissue samples (Figure 3F, P < 0.05), and miR-376a levels inversely correlated with *TTN-AS1* expression (Figure 3G; r = -0.6751, P < 0.0001). In addition, the expression of miR-376a was evaluated in *TTN-AS1*–deficient HOS and MG-63 cells. The *TTN-AS1* knockdown remarkably upregulated miR-376a in HOS and MG-63 cells (Figure 3H, P < 0.05). Overall, these results meant that *TTN-AS1* functions as a ceRNA (molecular sponge) for miR-376a in OS cells.

### MiR-376a acts as a tumor-suppressive miRNA in OS cells

**Figure 4:**
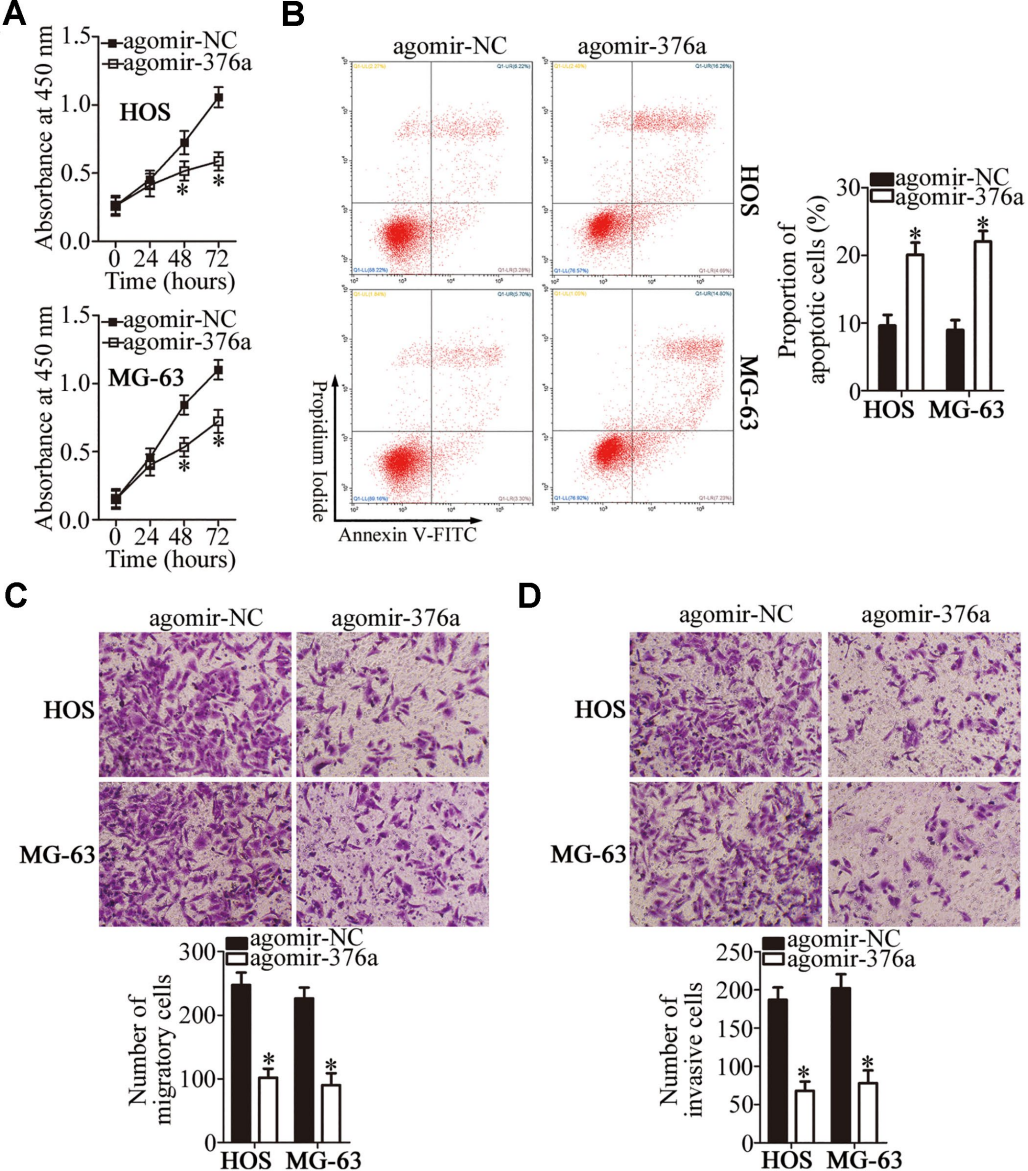
**MiR-376a exerts a tumor-suppressive action on the growth and metastasis of HOS and MG-63 cells.** (**A**, **B**) The CCK-8 assay and flow cytometry uncovered a change in proliferation and apoptosis of miR-376a-overexpressing HOS and MG-63 cells. *P < 0.05 vs. group agomir-NC. (**C**, **D**) HOS and MG-63 cells were treated with either agomir-376a or agomir-NC. After the transfection, Transwell migration and invasion assays were carried out. *P < 0.05 vs. group agomir-NC.

To examine the manner in which miR-376a regulates the malignancy of OS, the HOS and MG-63 cell lines were transfected with either agomir-376a or agomir-NC and, then, subjected to functional experiments. Transfection with agomir-376a appreciably decreased proliferation (Figure 4A, P < 0.05) and increased apoptosis (Figure 4B, P < 0.05) of HOS and MG-63 cells, as revealed by the CCK-8 assay and flow cytometry. Transwell migration and invasion assays showed that, when endogenous miR-376a was overexpressed, HOS and MG-63 cells had weaker migratory (Figure 4C, P < 0.05) and invasive abilities (Figure 4D, P < 0.05). In summary, these data suggested that miR-376a may suppress OS progression.

### *DKK1* mRNA is a direct target of miR-376a in OS cells

**Figure 5:**
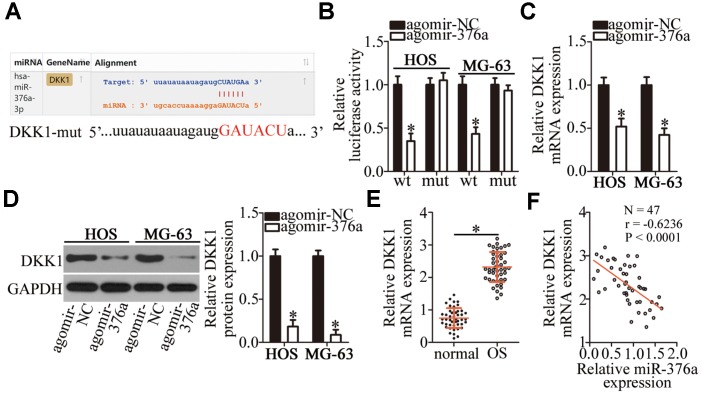
***DKK1* mRNA is a direct target of miR-376a in OS cells.** (**A**) MiR-376a and its wild-type binding site in the 3′-UTR of *DKK1* mRNA. The mutations were introduced into the site complementary to the seed region of miR-376a. (**B**) The luciferase reporter assay was performed to test whether the 3′-UTR of *DKK1* mRNA could be directly targeted by miR-376a in OS cells. HOS and MG-63 cells were cotransfected with either agomir-376a or agomir-NC and either the DKK1-wt or DKK1-mut plasmid. After 48 h of cultivation, the transfected cells were assayed with the Dual-Luciferase Reporter Assay System to measure the luciferase activity. *P < 0.05 vs. the agomir-NC group. (**C**, **D**) Expression levels of DKK1 mRNA and protein in miR-376a-overexpressing HOS and MG-63 cells were respectively determined by RT-qPCR and western blotting. *P < 0.05 vs. the agomir-NC group. (**E**) RT-qPCR was carried out to measure *DKK1* mRNA expression in the 47 pairs of OS tissue samples and adjacent-normal-bone tissue samples. *P < 0.05 vs. the normal bone tissues. (**F**) Spearman’s correlation analysis confirmed the negative correlation between *DKK1* mRNA and miR-376a levels among the OS tissues. r = -0.6236, P < 0.0001.

By means of target prediction tools, including starBase 3.0, TargetScan, and microRNA.org, *DKK1* was predicted as a potential target gene of miR-376a (Figure 5A). To validate this prediction, a luciferase reporter assay was performed on HOS and MG-63 cells after cotransfection with either agomir-376a or agomir-NC and either plasmid DKK1-wt (a plasmid expressing luciferase mRNA containing the *DKK1* 3′-UTR harboring a wild-type binding site for miR-376a) or plasmid DKK1-mut (a plasmid expressing luciferase mRNA containing the *DKK1* 3′-UTR harboring a mutated binding site for miR-376a). The ectopic expression of miR-376a significantly reduced the luciferase activity of DKK1-wt in HOS and MG-63 cells (P < 0.05). By contrast, mutation of the binding site abrogated this phenomenon (Figure 5B). Next, the expression levels of DKK1 in miR-376a-overexpressing HOS and MG-63 cells were determined to investigate whether DKK1 expression can be inhibited by miR-376a in OS. As expected, the mRNA (Figure 5C, P < 0.05) and protein levels (Figure 5D, P < 0.05) of DKK1 in HOS and MG-63 cells diminished in response to the agomir-376a transfection. To further characterize the correlation between miR-376a and DKK1 in OS, we quantified *DKK1* mRNA in the 47 pairs of OS tissue samples and adjacent normal bone tissues. DKK1 was found to be upregulated in the OS tissue samples in comparison with the adjacent normal bone tissues (Figure 5E, P < 0.05). The upregulation of *DKK1* mRNA negatively correlated with miR-376a levels among these OS tumor samples (Figure 5F; r = -0.6236, P < 0.0001). Collectively, the above findings identified *DKK1* mRNA as a direct target of miR-376a in OS cells.

### Restoration of DKK1 expression attenuates miR-376a-induced inhibition of the malignant characteristics of OS cells

**Figure 6:**
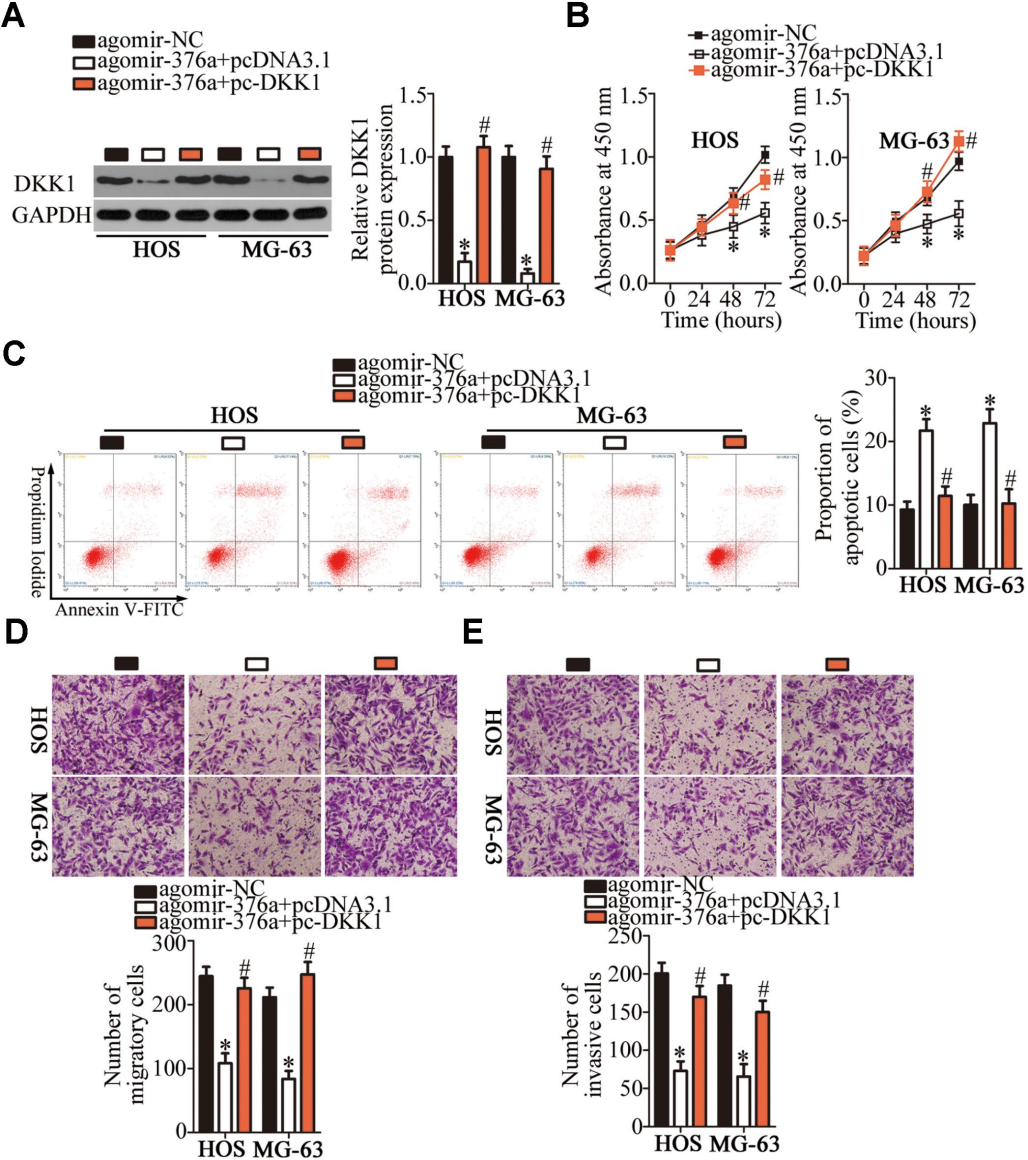
**DKK1 reintroduction attenuates miR-376a overexpression-mediated suppression of HOS and MG-63 cell proliferation, migration, and invasion as well as miR-376a overexpression-mediated promotion of apoptosis in vitro.** Agomir-376a was cotransfected with either plasmid pc-DKK1 or the empty pcDNA3.1 vector into HOS and MG-63 cells. (**A**) Total protein was isolated from the transfected cells and, then, subjected to western blotting for DKK1 protein quantification. *P < 0.05 vs. group agomir-MC. ^#^P < 0.05 vs. group agomir-376a+pcDNA3.1. (**B**–**E**) The proliferation, apoptosis, migration, and invasiveness of the aforementioned cells were analyzed by the CCK-8 assay, flow cytometry, and Transwell migration and invasion assays, respectively. *P < 0.05 vs. the agomir-MC group. ^#^P < 0.05 vs. group agomir-376a+pcDNA3.1.

Rescue experiments were conducted to confirm the involvement of DKK1 in the tumor-suppressive functions of miR-376a in OS cells. For this purpose, the DKK1-overexpressing plasmid lacking its 3′-UTR (pcDNA3.1-DKK1, hereafter: pc-DKK1) was introduced into miR-376a-overexpressing HOS and MG-63 cells. As presented in Figure 6A, the miR-376a overexpression-mediated decrease in DKK1 protein amounts almost disappeared in HOS and MG-63 cells after cotransfection with pc-DKK1 (P < 0.05). Subsequently, a series of experiments was performed on the HOS and MG-63 cells treated as described above. Restoration of DKK1 expression attenuated the effects of miR-376a overexpression on the proliferation (Figure 6B, P < 0.05), apoptosis (Figure 6C, P < 0.05), migration (Figure 6D, P < 0.05), and invasiveness (Figure 6E, P < 0.05) of HOS and MG-63 cells. These observations confirmed DKK1 downregulation as a functional mediator of miR-376a actions in OS cells.

### *TTN-AS1* enhances the malignant characteristics of OS cells in vitro through the miR-376a–DKK1 axis

**Figure 7:**
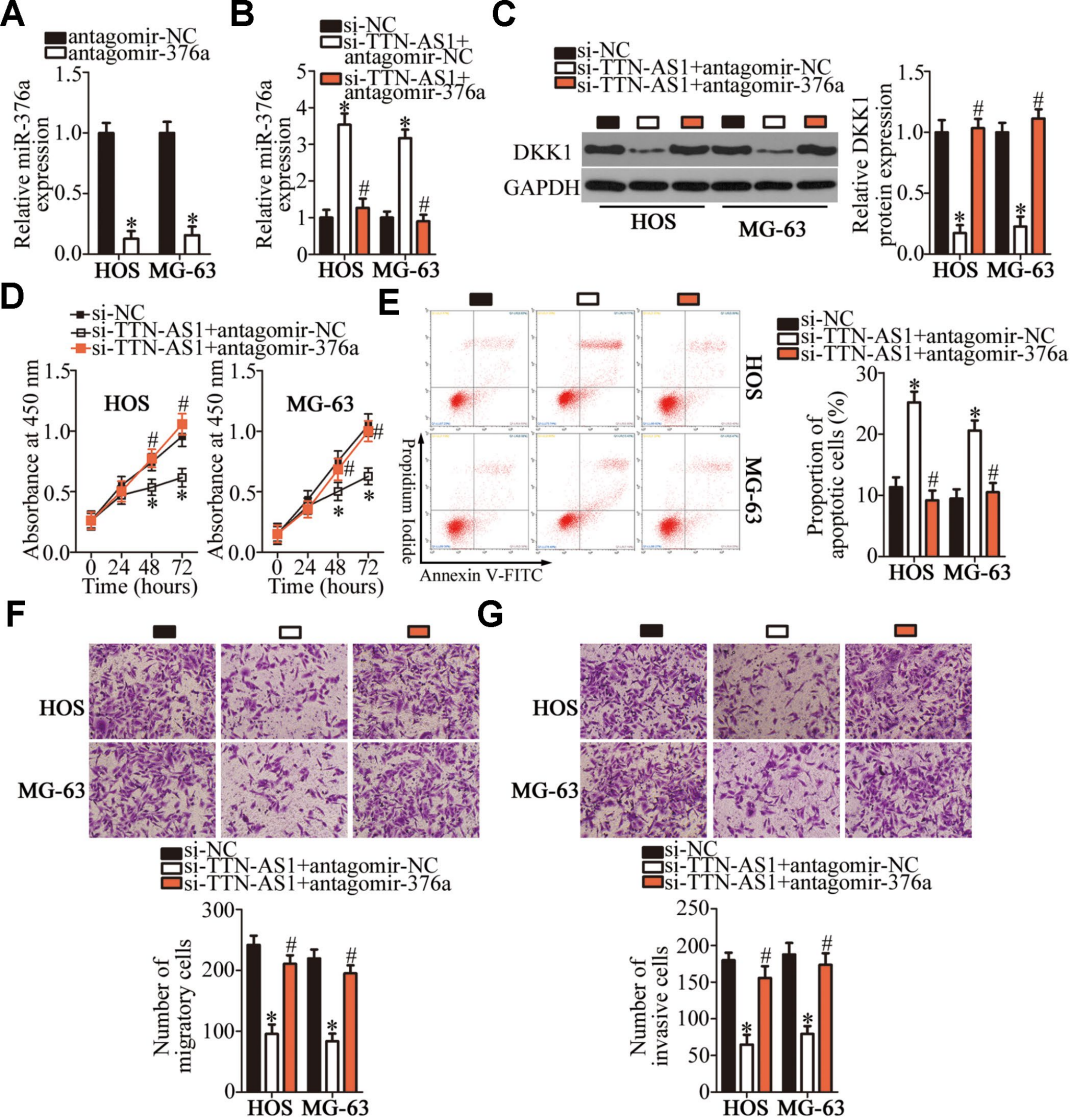
***TTN-AS1* enhances HOS and MG-63 cell proliferation, migration, and invasion and inhibits their apoptosis, via the miR-376a–DKK1 axis.** (**A**) Either antagomir-376a or antagomir-NC was introduced into HOS and MG-63 cells. The transfection efficiency was assessed through RT-qPCR. *P < 0.05 vs. the antagomir-NC group. (**B**, **C**) Si-TTN-AS1 in combination with either antagomir-376a or antagomir-NC was transfected into HOS and MG-63 cells. After 48 h transfection, expression levels of the DKK1 protein and miR-376a were determined respectively by western blotting and RT-qPCR. *P < 0.05 vs. group si-NC. ^#^P < 0.05 vs. group si-TTN-AS1+antagomir-NC. (**D**) The CCK-8 assay was conducted to evaluate the proliferative ability of HOS and MG-63 cells after cotransfection with si-TTN-AS1 and either antagomir-376a or antagomir-NC. *P < 0.05 vs. the si-NC group. ^#^P < 0.05 vs. group si-TTN-AS1+antagomir-NC. (**E**) The proportion of apoptotic HOS or MG-63 cells that were cotransfected with either antagomir-376a or antagomir-NC and si-TTN-AS1 was determined via flow cytometry. *P < 0.05 vs. the si-NC group. ^#^P < 0.05 vs. group si-TTN-AS1+antagomir-NC. (**F**, **G**) Transwell migration and invasion assays were conducted to evaluate the migratory and invasive abilities of HOS and MG-63 cells treated as described above. *P < 0.05 vs. the si-NC group. ^#^P < 0.05 vs. group si-TTN-AS1+antagomir-NC.

To test whether the oncogenic effects of *TTN-AS1* on the malignancy of OS cells were mediated by its influence on the miR-376a–DKK1 pathway, rescue experiments were performed on *TTN-AS1*-deficient HOS and MG-63 cells via transfection with miR-376a antagomir (antagomir-376a). First, the transfection efficiency of antagomir-376a was assessed by RT-qPCR. MiR-376a expression was found to be efficiently silenced in HOS and MG-63 cells after the transfection with antagomir-376a (Figure 7A, P < 0.05). The increase in miR-376a expression (via agomir-376a; Figure 7B, P < 0.05) and the downregulation of the DKK1 protein (Figure 7C, P < 0.05) caused by the *TTN-AS1* knockdown in HOS and MG-63 cells were partially reversed after cotransfection with antagomir-376a. Furthermore, the recovery of miR-376a and DKK1 expression counteracted the impact of the *TTN-AS1* knockdown on the proliferation (Figure 7D, P < 0.05), apoptosis (Figure 7E, P < 0.05), migration (Figure 7F, P < 0.05), and invasiveness (Figure 7G, P < 0.05) of HOS and MG-63 cells. These data revealed that the *TTN-AS1* knockdown reduced the malignancy of OS cells in vitro by decreasing the sponging of miR-376a by *TTN-AS1* and, thereby, reducing DKK1 expression.

### The *TTN-AS1* knockdown inhibits the in vivo tumor growth of OS cells

**Figure 8:**
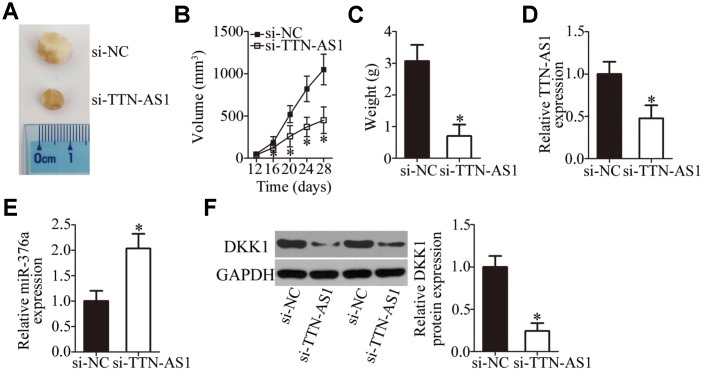
***TTN-AS1* downregulation restricts tumor growth of OS cells in vivo.** (**A**) Representative images captured at 4 weeks after subcutaneous injection of si-TTN-AS1-transfected or si-NC-transfected HOS cells into the flank of nude mice. (**B**) Tumor growth curves show that tumors grew significantly more slowly in the si-TTN-AS1 group than in the si-NC group. *P < 0.05 vs. group si-NC. (**C**) The average weight of tumor xenografts collected in groups si-TTN-AS1 and si-NC was analyzed at 4 weeks after the inoculation. *P < 0.05 vs. the si-NC group. (**D**, **E**) The expression levels of *TTN-AS1* and miR-376a in the tumor xenografts were measured by RT-qPCR. *P < 0.05 vs. group si-NC. (**F**) DKK1 protein expression was measured in the tumor xenografts by western blotting. *P < 0.05 vs. the si-NC group.

Xenograft tumors were induced to test whether there is a similar influence of *TTN-AS1* on tumor growth in vivo as in the above experiments in vitro. HOS cells transfected with either si-TTN-AS1 or si-NC were subcutaneously injected into a flank of nude mice. At 28 days postinoculation, the tumor growth curve indicated that the growth of tumor xenografts was much slower in the si-TTN-AS1 group than in the si-NC group (Figure 8A and 8B, P < 0.05). At the end of this experiment, all the mice were euthanized and the tumor xenografts were excised and weighed. The weight of the tumor xenografts derived from si-TTN-AS1–transfected HOS cells was obviously lower (Figure 8C, P < 0.05). Additionally, *TTN-AS1* expression was still low in the tumor xenografts from the si-TTN-AS1 group (Figure 8D, P < 0.05). The expression of miR-376a was higher (Figure 8E, P < 0.05), whereas the protein expression of DKK1 was lower (Figure 8F, P < 0.05), in the si-TTN-AS1 group than in the si-NC group. These results indicated that the downregulation of *TTN-AS1* retarded the in vivo tumor growth of OS cells by decreasing the output of the miR-376a–DKK1 axis.

## DISCUSSION

To date, numerous studies have revealed alterations in the expression of lncRNAs in OS [32–34]. The dysregulation of lncRNAs is proven to strongly influence the initiation and progression of OS by playing either a tumor-suppressive or oncogenic role [35]. Therefore, determination of the specific functions of lncRNA in OS is urgently needed for the identification and validation of novel diagnostic biomarkers of (and therapeutic targets in) OS. In this study, we first tested whether *TTN-AS1* is dysregulated in OS and clarified the clinical value of *TTN-AS1*. Second, we used siRNA to knock down endogenous *TTN-AS1* in OS cell lines, to study the influence of this *TTN-AS1* knockdown on the malignant characteristics of OS cells in vitro and in vivo. Third, the mechanisms underlying the participation of *TTN-AS1* in OS were elucidated in detail.

*TTN-AS1* is overexpressed in cervical cancer, and its upregulation obviously correlates with the International Federation of Gynecology and Obstetrics (FIGO) stage, poor differentiation, and lymph node metastasis [24]. Patients with cervical cancer overexpressing *TTN-AS1* have worse clinical outcomes than patients with cervical cancer underexpressing *TTN-AS1* [24]. The expression of *TTN-AS1* is high in papillary thyroid cancer, implying a close association with lymphatic metastasis, TNM stage, and overall survival [25]. Additionally, *TTN-AS1* is overexpressed in gastric cancer tissues and cell lines; this overexpression negatively correlates with overall survival of these patients [26]. Moreover, the overexpression of *TTN-AS1* is implicated in many tumor types including hepatocellular carcinoma [27], esophageal squamous cell carcinoma [28], and lung adenocarcinoma [29, 30]. Nevertheless, the expression of *TTN-AS1* in OS has not yet been studied. In this work, we found that *TTN-AS1* is upregulated in both OS tumors and cell lines. *TTN-AS1* overexpression significantly correlated with the clinical stage and distant metastasis among the patients with OS. Notably, the patients with OS overexpressing *TTN-AS1* showed substantially shorter overall survival compared to the patients with OS underexpressing *TTN-AS1*. These observations suggest that *TTN-AS1* may be an effective biomarker for the diagnosis and prognosis of OS.

*TTN-AS1* has been identified elsewhere as an oncogenic lncRNA during carcinogenesis and cancer progression. For instance, silencing of *TTN-AS1* expression inhibits cervical cancer cell proliferation and invasion both in vitro and in vivo by regulating the miR-573–E2F3 pathway [24]. In papillary thyroid cancer, *TTN-AS1* serves as a ceRNA for miR-155-3p and facilitates the expression of zinc and ring finger 2, thus, promoting cellular proliferation, migration, invasion, and epithelial–mesenchymal transition [25]. *TTN-AS1* promotes the proliferation, migration, invasiveness, and epithelial–mesenchymal transition of esophageal squamous cell carcinoma cells by acting as a ceRNA on miR-133b, thereby, increasing fascin 1 expression [28]. Besides, *TTN-AS1* promotes the malignancy of gastric cancer [26], hepatocellular carcinoma [27], and lung adenocarcinoma [29, 30]. In contrast, studies on the detailed functions of *TTN-AS1* in OS are rare. In this study, we found that the *TTN-AS1* knockdown restricted the proliferation, migration, and invasiveness of OS cells in vitro; increased their apoptosis in vitro; and slowed their tumor growth in vivo.

Subsequently, we elucidated the molecular mechanisms underlying the oncogenic actions of *TTN-AS1* in OS. In recent years, studies revealed that lncRNAs perform their functions by acting as ceRNAs or molecular sponges to inactivate miRNAs. Herein, our data provide reliable evidence that *TTN-AS1* serves as a ceRNA for miR-376a. MiR-376a is frequently reported to be upregulated in ovarian cancer [36] and downregulated in renal cell carcinoma [37], gastric cancer [38], and breast cancer [39]. MiR-376a is underexpressed in OS tumors and cell lines [40]. Its upregulation inhibits OS cell proliferation and invasion in vitro but promotes apoptosis [40]. In terms of the mechanism, F-box protein 11 mRNA is proved to be a direct target of miR-376a in OS [40]. Our results on the expression and functions of miR-376a in OS are consistent with these observations. Another important finding of this study is that the tumor-suppressive activity of miR-376a in OS is due to DKK1 downregulation. DKK1, a key factor in bone metabolism and homeostasis, is closely related to the malignancy of OS and helps to maintain or increase cellular proliferation, colony formation, metastasis, and tumorigenesis while reducing apoptosis [41–44]. Consequently, targeting the *TTN-AS1*–miR-376a–DKK1 pathway might be an innovative strategy to treat patients with OS.

In conclusion, we demonstrated that *TTN-AS1* is overexpressed in OS and associated with poor clinical outcomes. Moreover, this study is the first to reveal that *TTN-AS1* enhances the malignant characteristics of OS cells in vitro and in vivo. As for the mechanism, for the first time, *TTN-AS1* is reported here to act as a ceRNA on miR-376a, thereby, upregulating *DKK1* in OS. These findings may offer a novel theoretical and experimental explanation for OS progression and should help to find attractive therapeutic targets in OS.

## METHODS

### Ethics statement

Investigation has been conducted in compliance with the principles of the Declaration of Helsinki and according to national and international guidelines and has been approved by the authors' institutional review board. The study protocol was approved by the Ethics Committee of Liaoning Cancer Hospital & Institute. Informed consent has been obtained from all patients.

### Clinical tissues and cell lines

Primary OS tissue samples and adjacent normal bone tissues were obtained from 47 patients who underwent surgical resection at the Liaoning Cancer Hospital & Institute. None of the patients had received preoperative chemotherapy, radiotherapy, or other anticancer modalities. After the resection, the tissue samples were immediately frozen in liquid nitrogen and, then, stored in liquid nitrogen until subsequent treatment and analysis.

Four OS cell lines—HOS, SAOS-2, MG-63, and U2OS—as well as normal osteoblasts (hFOB1.19 cells) were bought from the Shanghai Institute of Biochemistry and Cell Biology (Shanghai, China). All the cells were cultured at 37°C in a humidified atmosphere containing 5% of CO_2_ in Dulbecco’s Modified Eagle’s Medium (DMEM) supplemented with 10% of fetal bovine serum (FBS),100 U/ml penicillin, and 100 μg/ml streptomycin (all from Gibco, Invitrogen Life Technologies, Carlsbad, CA, USA).

### Oligonucleotides, construction of plasmids, and cell transfection

The siRNA specifically decreasing the expression of *TTN-AS1* (called si-TTN-AS1) and its negative control siRNA (si-NC) were designed and commercially synthesized by RiboBio Co., Ltd. (Guangzhou, China). Agomir-376a, the corresponding agomir-NC, antagomir-376a, and antagomir-NC were acquired from the GenePharma Co., Ltd. (Shanghai, China). The full-length *DKK1* sequence lacking its 3′-UTR was amplified by the GenePharma Co., Ltd., too, and subcloned into the pcDNA3.1 vector to generate the pc-DKK1 plasmid. The empty pcDNA3.1 vector served as the control. Cells were seeded in 6-well plates and transfected with the agomir (50 nM), antagomir (50 nM), siRNA (100 pmol) or plasmid (4 μg) using the Lipofectamine® 2000 reagent (Invitrogen; Thermo Fisher Scientific, Inc.). The transfected cells were processed for further in vitro experiments after incubation for different periods.

### RNA isolation and RT-qPCR

The TRIzol® Reagent (Invitrogen; Thermo Fisher Scientific, Inc.) was used to isolate total RNA form tissue samples or cells. The miScript Reverse Transcription Kit was purchased from Qiagen GmbH (Hilden, Germany) and, then, used for the synthesis of cDNA from the total RNA. After reverse transcription, qPCR was performed using the miScript SYBR Green PCR Kit (Qiagen GmbH) to determine miR-376a expression. This expression was determined in relation to U6 small nuclear RNA. To quantitate *TTN-AS1* and *DKK1* expression, the synthesis of cDNA was performed using the PrimeScript RT-Reagent Kit (Takara Bio, Kusatsu, Japan), followed by qPCR with the SYBR Premix Ex Taq™ Kit (Takara Bio). Expression levels of *TTN-AS1* and *DKK1* were normalized to *GAPDH*. All the data were analyzed by the 2^−ΔΔCq^ method.

### CCK-8 assay

In 96-well plates, after 24 h transfection, cells were maintained in 10% FBS-supplemented DMEM for 0, 24, 48, or 72 h. At every time point, 10 μl of the CCK-8 solution (Dojindo Laboratories, Kumamoto, Japan) was added into each well, and the cells were incubated further at 37°C for 2 h. Optical density was measured at 450 nm wavelength on a Sunrise™ microplate reader (Tecan Group, Ltd., Mannedorf, Switzerland).

### Apoptosis measurement by flow cytometry

Transfected cells in 6-well plates were harvested at 48 h post-transfection. Next, they were washed in ice-cold phosphate-buffered saline and the apoptosis rate was analyzed via the Annexin V–Fluorescein Isothiocyanate (FITC) Apoptosis Detection Kit (Biolegend, San Diego, CA, USA). The transfected cells were resuspended in 100 μl of Annexin-V-binding buffer that was supplemented with 5 μl of Annexin V–FITC and 5 μl of a propidium iodide solution. After 15 min incubation at room temperature in the dark, the proportion of apoptotic cells was determined on a flow cytometer (FACScan; BD Biosciences, Franklin Lakes, NJ, USA).

### Transwell migration and invasion assays

Cell migratory capacity was analyzed in Transwell chambers (8.0 μm pore size; BD Biosciences, Franklin Lakes, NJ, USA), whereas Matrigel (BD Biosciences)-coated Transwell chambers were used for assessing cell invasiveness. Briefly, 200 μl of a cell suspension containing 5 ×10^4^ cells was added to the top chambers. The lower compartments were covered with DMEM supplemented with 20% of FBS, which served as the chemoattractant. After 24 h incubation, the nonmigratory cells and noninvasive cells were gently removed with cotton swabs. The cells located on the lower side of the chamber were fixed in 4% paraformaldehyde and stained with 0.5% crystal violet. Finally, the migratory and invasive abilities were assessed via counting of the respective migratory and invasive cells in five randomly selected visual fields in images that were captured by a CKX41 inverted microscope (Olympus Corp., Tokyo, Japan).

### Tumor xenograft experiment

A total of 8 six-week-old female BALB/c nude mice were purchased from Better Biotechnology Co., Ltd. (Nanjing, China). All the animal procedures were approved by the Institutional Animal Care and Use Committee of Jilin University and carried out in compliance with the Animal Protection Law of the People’s Republic of China-2009 for experimental animals. Si-TTN-AS1-transfected or si-NC-transfected HOS cells (1 × 10^7^) were resuspended in 100 μl of phosphate-buffered saline and inoculated subcutaneously into the flank of nude mice (n=4 for each group). The tumor size was recorded every 4 days, and tumor volume was calculated using the following formula: tumor volume = 1/2 × tumor length × tumor width^2^. After 4 weeks, all the mice were euthanized by cervical dislocation, and their tumor xenografts were excised and weighed.

### Nuclear/cytoplasmic fractionation

The PARIS Kit (Invitrogen; Thermo Fisher Scientific, Inc.) was used to isolate the cytoplasmic and nuclear fractions.

### RIP assay

The binding of miR-376a to *TTN-AS1* was assessed by means of the Magna RIP RNA-Binding Protein Immunoprecipitation Kit (Millipore Inc., Billerica, MA, USA). In brief, cells were incubated with RIP buffer containing magnetic beads coated with either the antibody to AGO2 or with control IgG. Subsequently, extraction of coprecipitated RNA was performed and the RNA was subjected to RT-qPCR analysis.

### Bioinformatics prediction and luciferase reporter assay

A target prediction tool, starBase 3.0 (http://starbase.sysu.edu.cn/), was used to search for potential miRNAs that could be inactivated by TTN-AS1. The target genes (mRNAs to be precise) of miR-376a were predicted by means of three miRNA target prediction databases: starBase 3.0, TargetScan (http://www.targetscan.org/), and microRNA.org (http://www.microrna.org/microrna/).

The DKK1 3′-UTRs containing either the wild-type (wt) binding sequence or the mutant (mut) binding sequence for miR-376a were synthesized by GenePharma Co., Ltd., and inserted into the pmirGLO luciferase reporter vector (Promega, Madison, WI, USA). The resultant plasmids are referred to as DKK1-wt and DKK1-mut, respectively. The TTN-AS1-wt and TTN-AS1-mut reporter plasmids were generated by similar experimental procedures. Either agomir-376a or agomir-NC was cotransfected into cells with either a “wt” or “mut” reporter plasmid using the Lipofectamine® 2000 reagent. Luciferase activity was determined 48 h after the transfection using a Dual-Luciferase Reporter Assay System (Promega, Madison, WI, USA). *Renilla* luciferase activity was normalized to that of firefly luciferase.

### Western blotting analysis

Total protein was isolated by means of the RIPA buffer (Beyotime Institute of Biotechnology, Shanghai, China). The Bicinchoninic Acid Protein Assay Kit (Beyotime Institute of Biotechnology, Shanghai, China) was used to quantify the protein concentration. The protein samples were resolved by SDS-PAGE on a 10% gel and transferred onto polyvinylidene difluoride membranes, followed by 2 h blocking with 5% fat-free milk diluted in Tris-buffered saline containing 0.1% of Tween 20 (TBST). After incubation with a primary antibody against DKK1 (cat. No. ab109416; dilution 1:1,000; Abcam, Cambridge, UK) or against GAPDH (cat. No. ab181603; dilution 1:1,000; Abcam), the membranes were washed thrice with TBST, probed with a goat anti-rabbit immunoglobulin G antibody conjugated with horseradish peroxidase (cat. No. ab205718; dilution 1:5,000; Abcam) (secondary antibody) and, then, treated with the Pierce™ ECL Western Blotting Substrate (Pierce; Thermo Fisher Scientific, Inc.) for visualization of the protein signals.

### Statistical analysis

Data are expressed as the mean ± standard deviation (SD) of at least three independent experiments. The correlation between *TTN-AS1* expression and clinical parameters among the patients with OS was examined with the χ^2^ test. Spearman’s correlation analysis was conducted to determine the correlation between *TTN-AS1* and miR-376a expression levels in OS tissue samples. The overall-survival curve was analyzed by the Kaplan–Meier method and compared between groups by the log-rank test. The comparisons between two groups were performed by Student’s *t* test; one-way analysis of variance, followed by the Student–Newman–Keuls test, was applied to evaluate the differences among multiple groups. Data with P < 0.05 were considered statistically significant.
